# Rapid Bacterial Motility Monitoring Using Inexpensive 3D-Printed OpenFlexure Microscopy Allows Microfluidic Antibiotic Susceptibility Testing

**DOI:** 10.3390/mi13111974

**Published:** 2022-11-14

**Authors:** Tai The Diep, Sarah Helen Needs, Samuel Bizley, Alexander D. Edwards

**Affiliations:** 1 Reading School of Pharmacy, University of Reading, Reading RG6 6AD, UK; 2 Capillary Film Technology Ltd., Billingshurst RH14 9TF, UK

**Keywords:** antibiotic motility test, antimicrobial resistance, microfluidic, bacterial cytometry, digital microscope, 3D printing

## Abstract

Antibiotic susceptibility testing is vital to tackle the emergence and spread of antimicrobial resistance. Inexpensive digital CMOS cameras can be converted into portable digital microscopes using 3D printed x-y-z stages. Microscopic examination of bacterial motility can rapidly detect the response of microbes to antibiotics to determine susceptibility. Here, we present a new simple microdevice-miniature microscope cell measurement system for multiplexed antibiotic susceptibility testing. The microdevice is made using melt-extruded plastic film strips containing ten parallel 0.2 mm diameter microcapillaries. Two different antibiotics, ceftazidime and gentamicin, were prepared in Mueller-Hinton agar (0.4%) to produce an antibiotic-loaded microdevice for simple sample addition. This combination was selected to closely match current standard methods for both antibiotic susceptibility testing and motility testing. Use of low agar concentration permits observation of motile bacteria responding to antibiotic exposure as they enter capillaries. This device fits onto the OpenFlexure 3D-printed digital microscope using a Raspberry Pi computer and v2 camera, avoiding need for expensive laboratory microscopes. This inexpensive and portable digital microscope platform had sufficient magnification to detect motile bacteria, yet wide enough field of view to monitor bacteria behavior as they entered antibiotic-loaded microcapillaries. The image quality was sufficient to detect how bacterial motility was inhibited by different concentrations of antibiotic. We conclude that a 3D-printed Raspberry Pi-based microscope combined with disposable microfluidic test strips permit rapid, easy-to-use bacterial motility detection, with potential for aiding detection of antibiotic resistance.

## 1. Introduction

The analysis of cellular behavior by cytometry can arguably provide insight into bacterial behavior more rapidly than most other methods. This can be useful for making quick phenotypic observations, including antibiotic susceptibility. Rapid and portable measurements of antibiotic susceptibility are needed to combat the increasing spread of antibiotic resistance. Bacterial motility can provide some information on bacterial identification, but can also be used to distinguish between live and dead cells and can be used as a measure of antibiotic susceptibility [1].

Motility has been widely acknowledged as a virulence factor in some pathogenic bacteria and plays a critical role in the formation of biofilm, which increases bacterial tolerance to antibiotics [2,3]. For the last several decades, different methods have been developed to differentiate and classify bacteria based on their motility. There are four kinds of different movement of bacteria: darting motility is presented by *Vibrio* species; active motility by *Salmonella*; sluggish motility by *Bacillus* and *Clostridia* genus; and thumbing motility by *Listeria*. Initial developments of bacterial motility tests included the use of culture medium and naked-eye observation, such as in the Craigie, J. (1993) tube method, which was then advanced by the inclusion of microscopic examination (e.g., the Wet mount method) [4]. Furthermore, these techniques have been enhanced by the use of dyes, including carbon fuchsin, safranin, and methyl blue or a novel single-tube agar-based technique for motility enhancement of *E. coli* O157:H7. Likewise, microscopy-based in situ growth assays have been developed [5,6].

Open source hardware has also emerged as a major trend in scientific research. Open source systems make scientific equipment much more accessible not only for research but for a wider range of users [7]. One powerful digital imaging platform makes use of Raspberry Pi hardware in research, offering low cost and accessibility; indeed, this was originally developed as an educational platform. Raspberry Pi hardware has been incorporated into commercial laboratory instruments (e.g., NuGenius by Syngene) and in many research laboratories for custom laboratory equipment. Many of these are based on imaging systems. One low-cost open source multi-fluorescence imaging system was developed using simply a Raspberry Pi computer and Pi camera [8]. In addition to being low cost and allowing fully programmable imaging, the flexible Raspberry Pi GPIO output pins simplify addition of robotic sample manipulation or lighting control alongside imaging. Thus, digital imaging capability can be combined with robotics, for example, open source 3D printer hardware motion systems can be used as a platform for Raspberry Pi imaging, such as the Raspberry Pi camera Open source Laboratory Imaging Robot (POLIR) that consists of a 3D-printed 3-axis frame moving a Raspberry Pi camera controlled by an Arduino microcontroller, previously used for time-resolved analytical microbiology, including measuring antimicrobial resistance in bacteria [9]. Another simple and cost-effective Raspberry Pi system provides controlled temperature and pressure monitoring systems [10]. Affordable 3D printing has likewise made it easier for the science and engineering community to create rapid prototypes of complex products, paving the way for faster and easier setup of a lab [11]. Small portable 3D printed structures combined with CMOS digital cameras are ideal for microscopy, illustrated by the OpenFlexure system [12] and by digital holographic microscope [13]. Open source hardware allow scientists to share, customise and improve their instrumentation alongside software, ensuring that technological advances in digital imaging can drive rapid scientific progress.

As digital cameras become cheaper, there has been a move from traditional laboratory instruments (e.g., based on film camera, photomultipliers, photodiodes, or CCD cameras) to consumer cameras offering a simplified approach to producing scientific imaging devices. The use of small CMOS cameras for digital microscopy is transforming the cost/performance of microimaging. For example, the Raspberry Pi Camera Module v2 is affordable yet still contains a high-quality CMOS image sensor (3280 × 2464 pixels), with a fixed focus lens that can be reversed and mounted far from the sensor to produce high resolution macro images. This system has been extensively adapted for digital microscopy [14,15,16] by combination with 3D printed x-y-z stages. Matching this trend in microelectronics, recently, microfluidics-based analytical microbiology and diagnostic technology has emerged to offer a promise of improved healthcare systems by allowing the development of low-cost, rapid point-of-care diagnostic devices [17]. Ideally, microelectronics, such as small digital cameras, can be combined with microfluidics to perform diagnostic assays, such as immunoassays [18].

Here, we explore for the first time if the OpenFlexure microscope has the resolution to be used for microfluidic microbiology, to directly observe bacterial behaviour and motion. In this study, we combined inexpensive portable components for microbial cytometry, to establish the feasibility of rapidly monitoring bacterial motility in the presence of antibiotics. A novel device is presented that exploits melt-extruded microcapillary film to create panels of microfluidic antibiotic-loaded chambers [19]. We investigated whether the 3D-printed OpenFlexure microscope using a low-cost Raspberry Pi v2 camera has sufficient magnification and resolution to monitor bacterial motility in microdevices. Adequate magnification and contrast were achieved to view motile bacteria and allowed differences in behavior to be observed in the presence of antibiotics above the organisms’ minimum inhibitory concentration (MIC) for that antibiotic. We suggest that this combination of inexpensive mass-manufactured microfluidic devices with open source 3D-printed digital microscopes permits rapid bacterial cell measurement suitable for portable analytical microbiology tests, such as rapid antibiotic susceptibility testing.

## 2. Materials and Methods

### 2.1. Open Source 3D Printed Digital Microscope

The open flexure microscope was built following 3D designs and assembly instructions provided by the open flexure project with details available on a public repository (https://github.com/rwb27/OpenFlexure_microscope (accessed on 10 January 2021)) and the project website (https://openflexure.org/ (accessed on 10 January 2021)). Components were printed as advised, on a Prusa i3 MK2S (Prusa Research, Prague, Czech Republic) from standard polylactic acid (PLA; Ooznest, Essex UK). The camera was run using a Raspberry Pi (RPi) 3 Model B or Raspberry Pi 4 Model B single-board computer, operated by USB mouse and keyboard, focused manually with preview images displayed on a computer screen via HDMI. Python code scripts using the PiCamera library (https://picamera.readthedocs.io/ (accessed on 10 January 2021)) were used to control length of time to record videos, using default resolution at 15 frames per second.

### 2.2. Microbiology Materials and Methods

Isolates *E. coli* ATCC 25,922 (EC), *P. aueruginosa* ATCC 10,145 (PSA) and *K. pneumoniae* ATCC 13,883 (Klebsiella) strains were obtained from the National Collection of Type Cultures (NCTC, Salisbury, UK) maintained and subcultured on Luria Bertani (LB) agar and broth (ThermoFisher, Loughborough, UK) and serially diluted (1:10) to an appropriate cell density before testing for motility. Gentamicin, ceftazidime and triphenyl tetrazolium chloride (TTC) were purchased from Sigma Aldrich (Gillingham, UK). The microcapillary film (MCF), manufactured by Lamina Dielectrics Ltd. (Billlingshurst, West Sussex, UK) [20,21], with capillary diameter of 200 µm was used for this study.

### 2.3. Antibiotic-Loaded Microfluidic Test Strips

Capillaries were filled with warm liquid agar (0.4% *w*/*v*) in Mueller-Hinton broth containing various antibiotics, which was allowed to solidify in the capillaries. Twenty-millimeter-long strips were held in a 3D-printed holder with design files available at the project Zenodo repository (https://zenodo.org/record/7249682#.Y3yG8HbMJPY, accessed on 10 January 2021). Overnight cultures inoculated in LB broth were diluted to approximately 10^8^ CFU/mL, and the motility was compared to a non-antibiotic control and scored by migration distance along the microcapillaries after exposure to the antibiotics.

The novel microfluidic method was compared to a conventional multiwall stab method. To prepare semi-solid agar, Mueller-Hinton agar (0.4% *w*/*v*) was made and when cooled to below 55 °C, but before gelation, antibiotics were added with a dilution range between 0.0125 and 32 mg/mL (depending on the antibiotic), and 200 µL was added in each 96-well microtitre plate strip-well. TTC dye was added at 1% *w/v* to stain bacteria (Sigma, Gillingham, UK). Then, 10 µL of liquid overnight bacterial culture was stabbed into the surface of the agar with a 20 µL pipette tip, and strips were incubated at 37 °C for 16–18 h in the PiRamid time-lapse imaging box [Long et al., HardwareX, manuscript accepted]. Bacterial visible stab line and cloudiness were recorded as positive motile, and is considered resistant if antibiotic is present. In contrast, the visible stab line with clear agar was recorded as negative and thus non–motile, which was considered susceptible.

## 3. Results and Discussion

### 3.1. Concept of Microfluidic Bacterial Cytometry using Microcapillaries Combined with 3D-Printed Microscope

Microfluidic devices allow portable testing, transporting laboratory instruments into near-patient devices, and have been demonstrated for use of antibiotic susceptibility testing (AST). Motility and single-cell imaging provides one of the fastest measurements of phenotypic AST, providing results in as little as 20 min. However, these often require complex and bulky imaging or cytometry systems, such as microscopes or flow cytometers that require a laboratory environment to be operated. Previously, we have demonstrated the use of microcapillary film as a scalable and accurate AST device suited for point-of-care (PoC) applications, using a colour-based indicator to determine growth after several hours [19]. However, using single-cell imaging, there is a potential to simplify sample preparation and decrease the time to result.

**Figure 1:**
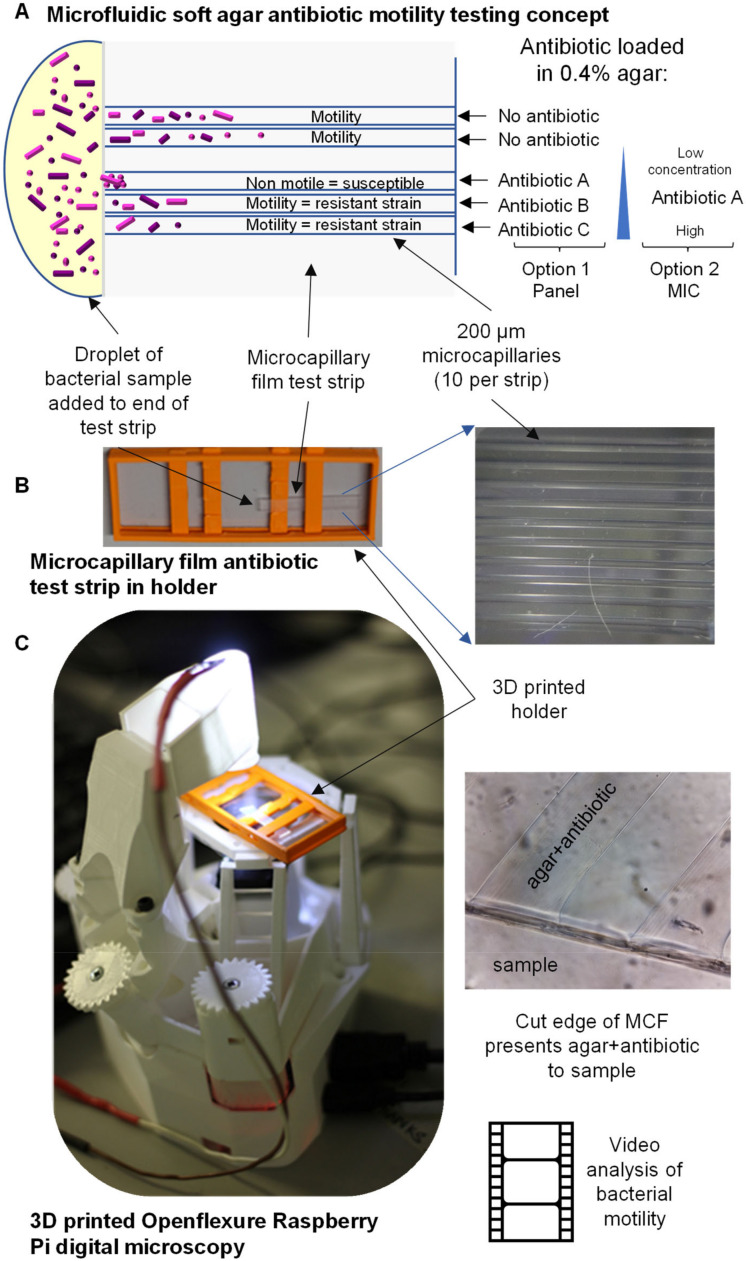
Combining inexpensive microfluidics with 3D-printed digital microscopy to create a simple cytometer for observing response of motile bacteria to antibiotics. (**A**) Schematic of configuration of microcapillary film strip presenting different antibiotics, or different concentrations of a single antibiotic, to a bacterial sample. (**B**) Test strip is contained in simple holder with transparent base; each holder can present four strips each of ten conditions to a single drop of sample. (**C**) Combined with 3D-printed OpenFlexure microscope allows direct observation of how motile bacterial cells response to exposure to antibiotics.

Here, we miniaturised a soft agar motility assay into microcapillaries (Figure 1). Samples containing bacteria are added to the open end of a microcapillary film test strip, with the capillaries containing soft agar with or without antibiotics. Motile bacteria are able to swim freely into the capillary end, and into the soft agar. However, if susceptible to the antibiotic, a change in motility was observed, including decreased migration into the capillary and after time, bacteria killed from antibiotic exposure stopped moving.

This test device was combined with a 3D-printed holder to fit the stage of the 3D-printed OpenFlexure microscope to record videos and thereby determine motility. In this way, a small and compact bacterial cytometer can be produced for under $200 (based on $100 for the Raspberry Pi computer, $30 for the v2 camera and a small budget for 3D printing and accessory parts). The 3D-printed OpenFlexure microscope is much smaller and lighter than a conventional laboratory microscope. The system is controlled by a Raspberry Pi, and the sample is lit by a single LED, allowing the system to be powered by 5V USB or even by battery rather than requiring mains power, making it ideal for field use or low-resource settings.

### 3.2. Establishing if System Has Sufficient Resolution to Observe Bacteria for Motility Assays

There is a trade-off between cost of instrument, field of view and resolution. Biological samples often use phase contrast microscopy or differential interference contrast (DIC) microscopy, allowing unstained samples and cellular structures to be more visible. The OpenFlexure lack these modifications, and the sample is lit simply from above using a single white LED. This provides limited contrast for cellular samples, yet the OpenFlexure microscope was originally developed for water quality testing [12].

**Figure 2:**
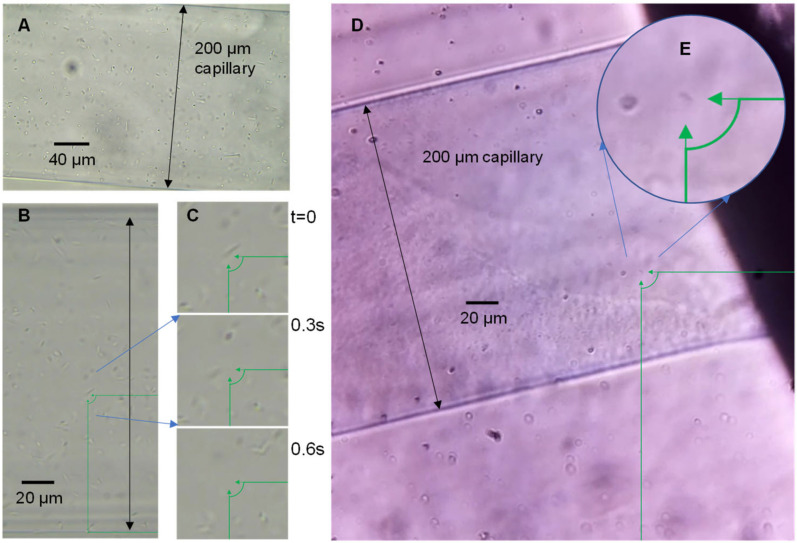
Comparison of conventional phase contrast microscopy (using 40X objective) with OpenFlexure 3D-printed digital microscope. Samples of *P. aeruginosa* are shown imaged using (**A**–**C**) conventional microscope compared with (**D**,**E**) OpenFlexure microscope. (**B**,**C**) show how the rapid movement of motile bacteria are clearly visible when the micrograph is enlarged. Although the OpenFlexure micrograph (**D**) has lower magnification, and bacterial cells are still clearly visible (**E**) but are smaller and with less intense contrast than the conventional microscope.

Imaging motility does not necessarily require high resolution or clear structural images; however, to fully observe sample motility and migration into the capillaries, the entire capillary opening needs to be observed. To identify if single bacteria could be imaged using the OpenFlexure microscope in the microcapillaries, a sample of *P. aeruginosa* was loaded into the devices and imaged on a conventional laboratory microscope equipped with a digital camera, and these were compared with the OpenFlexure system (Figure 2). At magnification in which the full diameter of the 200 µm diameter capillary is in view, individual *P. aeruginosa* are clearly visible, and the motile rod-shaped bacteria move quickly in the capillary (Figure 2C). Images and videos collected by the OpenFlexure microscope also show clear individual bacteria (Figure 2D), and both systems can identify individual bacteria with the full capillary diameter in view. We conclude that the OpenFlexure microscope can be used to image single bacterial cells and screen motility in unstained samples within microfluidic devices.

The focal plane of the microscope is smaller than the diameter of the microcapillaries, and the fixed focal length of the raspberry pi camera means the system has to be adjusted correctly to ensure that motility of bacteria inside the capillaries are observed. The system was focused on the widest part of the capillary, but this can mean that bacteria move in and out of focus as they swim above and below the focal plane. The system may be improved using smaller diameter capillaries or devices with flatter channels, or with robotic z-stage added to the OpenFlexure microscope that can scan and image the full depth of the microdevice.

### 3.3. Confirming Antibiotic Susceptibility Can Be Tested by Conventional Motility Assays

**Figure 3:**
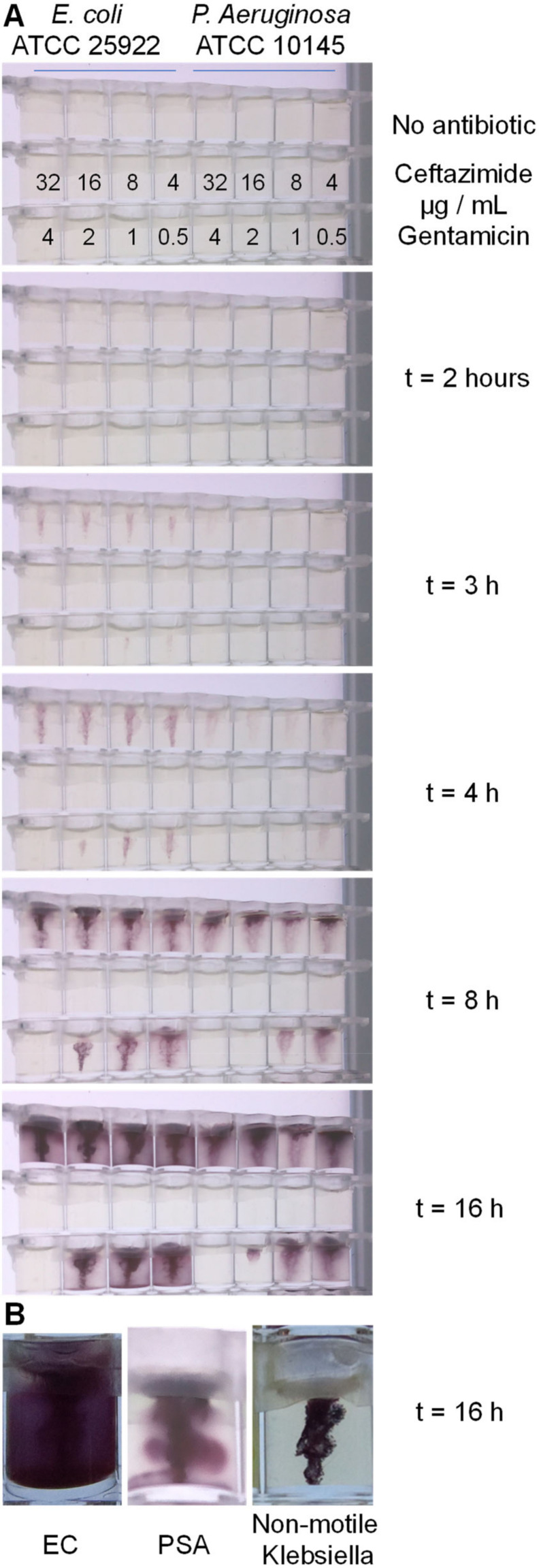
Confirming motility is inhibited by antibiotics using a combination of conventional methods. (**A**) Soft agar containing the indicated antibiotic concentrations was stabbed with an inoculum of the indicated motile bacteria, and time-lapse images recorded during overnight incubation. Dark staining by bacteria migrating away from the stab site become clear from 3 h, with spread across the whole well visible by 8–16 h; in contrast, antibiotics above the inhibitory concentration block migration and growth. (**B**) Close-up of endpoint images of motile *E. coli* and *P. aeruginosa* contrast with *K. pneumoniae.* Similar observations were seen in at least 5 independent experiments.

Motility assays are typically used to measure motility, and in contrast, different standardised antibiotic susceptibility testing (AST) methods, such as disc diffusion, broth microdilution or agar dilution, are used to measure antibiotic susceptibility [22]. We therefore checked whether motility measurement assays could be adapted to perform AST by combining conventional agar dilution AST with soft agar motility measurement. When checked using reference strains of *E. coli* and *P. aeruginosa*, this combination of conventional method allowed us to observe motility macroscopically with a range of antibiotic concentrations. Soft agar (0.4%) containing TTC and doubling dilutions of ceftazidime and gentamicin were poured into 96-well plate format microtitre stripwells. Cultures of *E. coli* and *P. aeruginosa* were stabbed into each well, and movement of bacteria away from the stab site was monitored during overnight incubation at 37 °C using Piramid, a time-lapse Raspberry Pi imaging box recently developed by our group [Long et al., HardwareX, accepted manuscript]. The MIC can be recorded as the lowest concentration of antibiotic that fully inhibits growth (Figure 3). The stab culture can clearly identify motile versus non-motile bacteria; the motile bacteria were able to swim into the soft agar, and live bacteria convert TTC, forming a diffuse bloom of purple dye. The reduction of TTC forms an insoluble dye that does not diffuse in the soft agar, making bacterial movement clearly recordable with the digital camera. In contrast, non-motile bacteria, such as *K. pneumoniae*, were still able to grow and convert the TTC, but the colour change is strictly limited to the original stab location (Figure 3B). The motile *E. coli* and *P. aeruginosa* isolates both showed clear spread without antibiotic, which was inhibited by antibiotics at expected concentrations. We observed an MIC of 4 µg/mL for gentamicin and <4 µg/mL for ceftazidime for both organisms. We conclude that conventional macroscopic methods demonstrate that a bacterial motility readout can be used to quantify antibiotic susceptibility.

### 3.4. OpenFlexure Microscope Plus Microcapillary Devices Allow Rapid Direct Microfluidic Bacterial Cytometry to Measure Antibiotics Inhibiting Motility

The feasibility of motility-based AST was then assessed in the low-cost, portable combined microfluidic cytometry system, with bacterial movement recorded directly at the inlet of microcapillaries using the OpenFlexure digital microscope. Results were recorded for two bacteria and two antibiotics at concentrations where motility was known to be inhibited in the soft agar-filled capillaries. Videos of 6 min duration were recorded using a frame-rate of 15 frames per second, which gave the best compromise between image resolution and stable video capture by the Raspberry Pi computer, resulting in movies that run at double speed of real-time microbial (Supplementary Materials listed in Table 1). The video files clearly show motile bacteria are able to migrate from the end along the capillary length in agar without antibiotics present (PSA no antibiotic) and that the distance travelled is affected by antibiotic.

The results of the microcapillary motility follow the same trends as the stab culture, although as is commonly a challenge for AST, the exact MIC is less clear cut and can be challenging to define. One aspect of this is due to timing. Motility was assessed after just 3 min, compared to 16 h under standard AST conditions. Furthermore, the test involved the bacteria moving into the soft agar capillaries to be exposed to the antibiotics; while motile bacteria can clearly migrate into soft agar, this process is likely to be slower than motility in liquid due to increased viscosity. One adjustment that could be made to this system would be to use liquid media cultures in capillaries containing dried antibiotics; this would expose the bacteria much faster to the antibiotic of interest. The disadvantage of such a liquid system is the risk of rapid mixing or flow of antibiotic solution out of the microcapillary, leading to less confidence in the antibiotic concentration. Another disadvantage of the agar-loaded antibiotic test strips is the limited stability of antibiotic in solution. Our test strips are unlikely to have long shelf-life for many antibiotics. However, the results presented here with this soft agar method clearly demonstrate the concept of low-cost compact 3D-printed microscope combined with melt-extruded microfluidic device

To measure motility in the 200 µm microcapillary film, individual bacteria need to be identifiable, as proven to be possible using the OpenFlexure microscope configured with an approximately 400–500 micron field of view. However, this is not possible with lower magnification that allows monitoring all ten capillaries at once, as the resolution is no longer enough to identify individual bacteria, so capillaries have to be monitored individually, and the stage moved to monitor the next capillary. This makes the system more involved to use, but this compromise between resolution and field of view is also true of conventional microscopes.

## 4. Conclusions

The OpenFlexure microscope provides a low-cost, portable microscopy that combined with increasingly more affordable portable devices, such as microfluidic systems, can allow field use cytometry, for example as near-patient diagnostics, in this example illustrated by rapid antibiotic resistance detection. The microscope has sufficient resolution to identify individual bacteria under simple single-LED bright field illumination, and video recording can readily capture the motility of unstained bacteria in the microcapillaries used in this study. The increase in availability of microelectronics such as low-cost but high-quality optoelectronics combined with microfluidics, will drive the development of new portable diagnostic devices, improving healthcare needs in especially in rural or low-resource settings.

## Figures and Tables

**Table 1:** Summary of motility observed in video recordings of microcapillaries with a range of antibiotic/bacteria combinations.

Video File Name ^#^	Organism	Antibiotic/Concentration	Motility Observed *
PSA no antibiotic	*P. aeruginosa* ATCC 10145	No antibiotic	+++
PSA Ceftazidime 0_5		Ceftazidime/0.5 µg^−1^	++
PSA Ceftazidime 4		Ceftazidime/4 µg^−1^	+
PSA Gentamicin 4		Gentamicin/4 µg^−1^	-
EC Gentamicin 0_5	*E. coli* ATCC 25922	Gentamicin/0.5 µg^−1^	+++
EC Gentamicin 4		Gentamicin/4 µg^−1^	+
EC Ceftazidime 32		Ceftazidime/32 µg^−1^	-

* Motility score based on migration distance along capillary: +++ >300 µm, ++300 < 201 µm, +200 < 100 µm, −99 < 0 µm. Similar observations were seen in at least five independent experiments. ^#^ Video files available from the project Zenodo repository at doi:10.5281/zenodo.7249682 or via URL https://zenodo.org/record/7249682 (accessed on 10 January 2021).

## Data Availability

Supporting data for this paper can be downloaded at: Rapid bacterial motility monitoring using inexpensive 3D-printed OpenFlexure microscopy|Zenodo.

## Supplementary Materials

The following supporting information can be downloaded at: https://zenodo.org/record/7249682, accessed on 30 October 2022. Rapid bacterial motility monitoring using inexpensive 3D-printed OpenFlexure microscopy|Zenodo, Video files: PSA no antibiotic, PSA Ceftazidime 0_5, PSA Ceftazidime 4, PSA Gentamicin 0_5, PSA Gentamicin 4, EC Gentamicin 0_5, EC Gentamicin 4, EC Ceftazidime 32.
